# Experiences of Lactation in GME: Are there Benefits Beyond the Parent and Infant?

**DOI:** 10.1007/s10995-025-04115-5

**Published:** 2025-06-19

**Authors:** Virginia Sheffield, Sarah Tomlinson, Harlan McCaffery, Amanda D. McCormick

**Affiliations:** 1 Department of Internal Medicine, Michigan Medicine, Ann Arbor, MI USA; 2 VA Ann Arbor Healthcare System, Ann Arbor, MI USA; 3 Department of Emergency Medicine, Michigan Medicine, Ann Arbor, MI USA; 4 Department of Pediatrics, Michigan Medicine, University of Michigan, 1500 E Medical Center Dr., Ann Arbor, MI 48109 USA

**Keywords:** Breastfeeding, Medical education, Lactation, Resident, Survey

## Abstract

**Introduction:**

Although lactating graduate medical education trainees often encounter barriers when returning to work—such as perceived challenges on clinical teams—the potential benefits arising from their lactation experiences remain understudied. In particular, no prior research has examined whether working alongside lactating trainees enhances knowledge and patient care. The purpose of this study was to assess trainee perceptions of how experiences with lactation impacted their knowledge of lactation and ability to care for lactating patients.

**Methods:**

In 2022, all residents and fellows at a large academic medical center were eligible to participate in an anonymous electronic survey. Data analyses included chi-squared testing of lactation experiences and trainee self-perceived ability to care for lactating patients.

**Results:**

133/1319 (10%) of trainees representing 31 programs completed the survey. 87% of participants disagreed that they felt uncomfortable about a co-resident pumping in their presence. Personal experience with lactation was associated with perceived increase in knowledge of lactation (*p* = 0.012) as well as perceived ability to better care for a lactating patient (*p* < 0.001) when compared with no experience with lactation, or experience through others. Among those without personal lactation experience, 71% felt their knowledge of lactation improved and 42% believed their ability to care for lactating patients was better due to their experiences working with lactating teammates.

**Conclusions:**

Lactation does not disrupt other residents on the team and may positively impact the care of lactating patients. The benefits of lactation may extend beyond the lactating parent and their infant.

## Significance

What is already known about this subject?

What this study adds?

Lactating graduate medical education trainees have significant challenges on returning to work, including safe and clean conditions to pump and impact of pumping on patient care, medical education, and perceived team dynamics.

A previous study showed that co-residents do not perceive their lactating colleagues to be disruptive to the team. This study explores perceptions on whether experiences with lactation, either personal or through someone else, improves knowledge and patient care. We found that many trainees reported improvement in knowledge and care of lactating patients after being exposed to lactation, with those with personal experience to most likely report these improvements.

## Introduction

As more individuals have children during medical training, it has become increasingly important to identify strategies for support upon return to work, address barriers to success, and examine the impact of lactation in the workplace (Blair et al., 2016). Prior studies have documented multiple difficulties faced by lactating residents at work (Ames & Burrows, 2019; Cantu et al., 2018; Dixit et al., 2015; Gupta et al., 2019; Peters et al., 2020). It has been shown that lactating residents feel that pumping can be detrimental to their job and adversely affect the team (Ames & Burrows, 2019). However, in that same study, co-residents of lactating residents did not report significant disruption to the team or patient care(Ames & Burrows, 2019). Little is understood about the experiences of trainees who work in proximity to lactating trainees and how this may impact patient care and co-resident perceptions of colleagues who pump at work. To our knowledge, no prior work has investigated the potential benefit to knowledge and patient care from working in proximity to lactating trainees. The purpose of this study was to assess trainee perceptions of how experiences with lactation impacted their knowledge of lactation and ability to care for lactating patients.

## Methods

In 2022, all residents and fellows at a large Midwest academic institution were eligible to participate in this cross-sectional study. Trainees were sent an anonymous electronic survey via Qualtrics. Survey responses were summarized using frequencies with percentages (%) or means with standard deviation for individual items. Chi-squared test was used to test associations between personal experience and proxy (through partners, coworkers, or friends) experience with lactation and perceived impact of being on teams with lactating residents on knowledge of lactation and being able to care for lactating patients.

The study was deemed exempt by the Institutional Review Board.

## Results

The survey was emailed to 1,319 graduate medical trainees and a total of 134 trainees (10%) representing 31 unique programs completed the survey. Demographics are summarized in Table 1. Of respondents, 42 (31%) reported having lactated and used a pump themselves, while 7 (5%) reported that their partner had done so. Additionally, 45 (34%) respondents reported being familiar with pumping from the experiences of close friends and 71 (53%) reported having worked in proximity with coworkers who pumped.

**Table 1:** Demographics by lactation experience (*N* = 134)

I have lactated and used a pump	No	Yes
	(*N* = 81)	(*N* = 42)
My partner has lactated and used a pump	No	Yes
		(*N* = 7)
Gender		
- Female	60 (74.1%)	42 (100.0%)
- Male	21 (25.9%)	0 (0.0%)
Specialty		
- Anesthesiology (General and Subspecialties)	10 (12.3%)	2 (4.8%)
- Dental	0 (0.0%)	2 (4.8%)
- Dermatology	1 (1.2%)	1 (2.4%)
- Emergency Medicine	5 (6.2%)	2 (4.8%)
- Family Medicine	5 (6.2%)	1 (2.4%)
- Internal Medicine (General and Subspecialties)	22 (27.2%)	15 (35.7%)
- Internal Medicine/Pediatrics	2 (2.5%)	0 (0.0%)
- Neurology	8 (9.9%)	3 (7.1%)
- No Response	1 (1.2%)	0 (0.0%)
- Pathology	4 (4.9%)	1 (2.4%)
- Pediatrics (General and Subspecialties)	2 (2.5%)	2 (4.8%)
- PM&R	2 (2.5%)	0 (0.0%)
- Psychiatry (General and Subspecialties)	9 (11.1%)	2 (4.8%)
- Radiology (General and Interventional)	0 (0.0%)	4 (9.5%)
- Surgery (General and Subspecialties)	10 (12.3%)	7 (16.7%)
Post Graduate Year in Training	3.5 ± 1.8	4.3 ± 1.8
Number of Children		
0	71 (87.7%)	0 (0.0%)
1	5 (6.2%)	27 (64.3%)
2	3 (3.7%)	13 (31.0%)
3+	2 (2.5%)	1 (2.4%)
No response	0 (0.0%)	1 (2.4%)
Age in Years	30.9 ± 2.9	31.8 ± 2.0

**Fig. 1:**
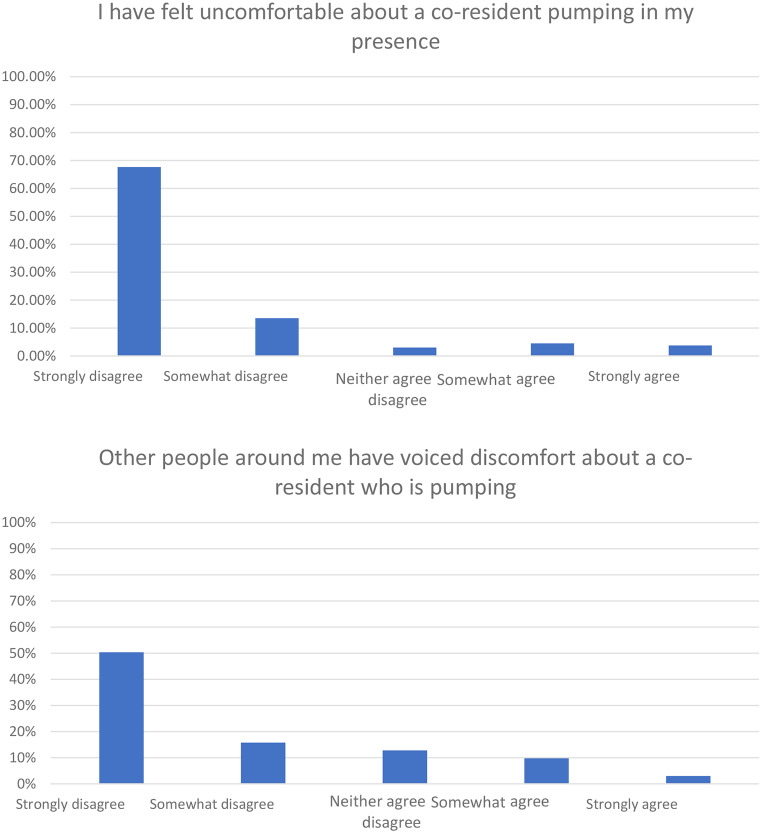
Results

Respondents were asked about their level of discomfort with a co-resident pumping in their presence as well as their perception of other people’s discomfort. Most respondents (*n* = 116, 87%) either strongly or somewhat disagreed with feeling uncomfortable about a co-resident pumping in their presence. When asked about the perceptions of others, 95 (71%) respondents strongly or somewhat disagreed that other people around them have voiced discomfort about a co-resident who is pumping (Fig. 1).

**Fig. 2:**
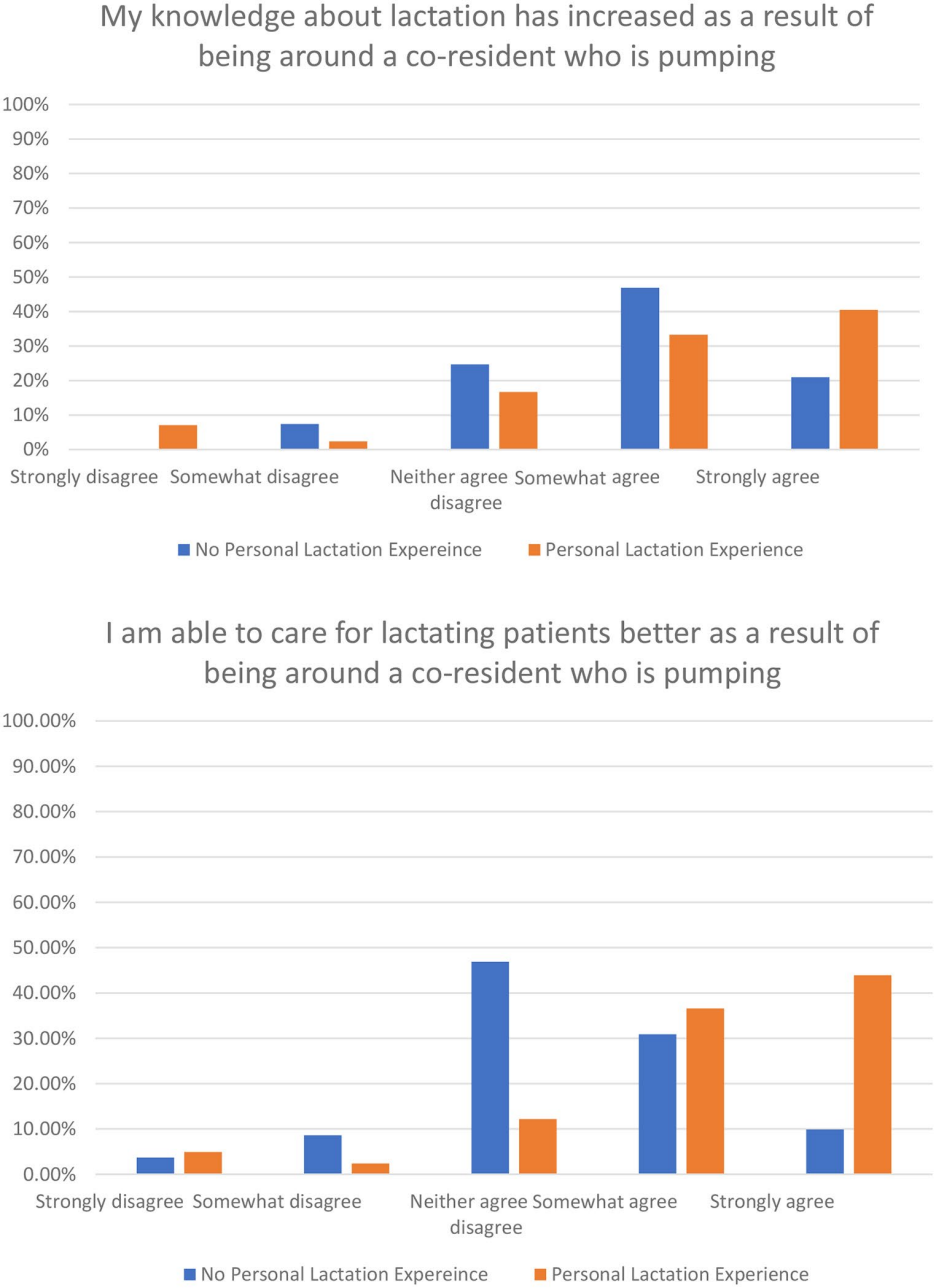
Impact of experiences with lactation on knowledge and patient care

Personal experience with lactation was associated with the level of perceived increase in knowledge of lactation (*p* = 0.012) as well as perceived ability to better care for a lactating patient (*p* < 0.001) when compared with no experience with lactation, or experience through others (Fig. 2). In both cases, those with personal experience with lactation had more “strongly agree” responses than any of the other responses (somewhat agree or below). For those without personal experience with lactation but who had worked on a team with lactating teammates, 71% reported perceived increase in knowledge of lactation as a result of being around lactating trainees, and 42% reported better perceived ability to care for a lactating patient as a result of being around lactating trainees.

## Discussion

To our knowledge, this is the first study evaluating the association between a trainee’s exposure to lactation and self-perceived ability to care for lactating patients. Our findings suggest that many trainees see a positive impact of exposure to lactation–either through their own lactation experiences or the experiences of others around them–on their ability to care for patients who are lactating. Similar to a prior study(Ames & Burrows, 2019), our study found that fewer than 10% of trainees reported discomfort related to lactating teammates.

In June 2018, the Accreditation Council for Graduate Medical Education (ACGME) Common Program Requirements approved revisions to instruct that residency and fellowship training sites provide appropriate support for space and time for lactation (Johnson et al., 2019). Policies alone will not adequately address the problems faced by lactating trainees, as true change will require a culture shift within academic medicine including “open culture of communication around lactation” (Pesch et al., 2019). This study reveals that exposure to co-trainees who are lactating can begin to spur this cultural shift. The existing literature focuses on the *personal* benefits of strong lactation support for the lactating individual’s wellness, and on potential *negative* impacts to other residents. This study, however, underlines the fact that the benefits of supporting lactating trainees extend beyond their personal wellness with increased co-trainee knowledge and may even result in improved patient care.

Results of this study must be interpreted with limitations in mind. Although the institution selected for this study is large with representation of many training programs, survey completion rate was low (10%). This may limit the generalizability of findings to the broader medical trainee population. Self-selection bias may also have impacted results, as the majority of our respondents were lactating residents. Additionally, those who chose to answer the study may be more likely to include those who have been more positively or more negatively impacted, and those with more neutral experiences may not feel compelled to complete the survey. The scope of our survey was narrow and may not fully encompass the entire experience of lactating residents. This study was conducted at a single center which may also limit generalizability of findings. Future studies at multiple institutions, across all specialties, and continuing to include trainees with and without personal lactation experience will best inform the impact of these experiences on the care of lactating patients.

## Data Availability

Given the cross-sectional survey nature of this study, full summary of the data is available in Table 1; Figs. 1 and 2, however data was not deposited.
